# Supplementary material for: Psychometric characteristics of the Hospital Anxiety and Depression Scale in stroke survivors of working age before and after inpatient rehabilitation
Source: PLoS One. 2024 Aug 26;19(8):e0306754. doi: 10.1371/journal.pone.0306754 (PMC11346913; doi:10.1371/journal.pone.0306754)
Supplement: S2 Fig — (DOCX) [file pone.0306754.s002.docx]

**S2 Fig.** Category probability curves showing response category functioning for the depression scale of the Hospital Anxiety and Depression Scale according to the Rasch partial credit model, at admission, discharge, and 1-year follow-up.

Admission Discharge 1-year follow-up

Admission Discharge 1-year follow-up

Admission Discharge 1-year follow-up

Admission Discharge 1-year follow-up

Admission Discharge 1-year follow-up

Admission Discharge 1-year follow-up

Admission Discharge 1-year follow-up
