# Supplementary material for: Psychometric characteristics of the Hospital Anxiety and Depression Scale in stroke survivors of working age before and after inpatient rehabilitation
Source: PLoS One. 2024 Aug 26;19(8):e0306754. doi: 10.1371/journal.pone.0306754 (PMC11346913; doi:10.1371/journal.pone.0306754)
Supplement: S1 Table — (DOCX) [file pone.0306754.s003.docx]

**S1 Table.** Stroke patients included in the analysis at hospital admission to inpatient rehabilitation (cohort 1), at discharge (cohort 2), and at 1-year follow-up (cohort 3).

| **Samples** | **Admission** | **Discharge** | **1-year follow-up** |
| --- | --- | --- | --- |
| **Cohort 1** | 256 | 147* | 70** |
| **Cohort 2** |  | 223 | 78*** |
| **Cohort 3** |  |  | 313 |

*Number of patients included in the sample both at admission and discharge

**Number of patients included in the sample both at admission and 1-year follow-up

***Number of patients included in the sample both at discharge and 1-year follow-up
