# Supplementary material for: Psychometric characteristics of the Hospital Anxiety and Depression Scale in stroke survivors of working age before and after inpatient rehabilitation
Source: PLoS One. 2024 Aug 26;19(8):e0306754. doi: 10.1371/journal.pone.0306754 (PMC11346913; doi:10.1371/journal.pone.0306754)
Supplement: S2 Table — (DOCX) [file pone.0306754.s004.docx]

**S2 Table.** Descriptive statistics for the anxiety and depression items of the Hospital Anxiety and Depression Scale (HADS) among

stroke patients at admission to inpatient rehabilitation, at discharge, and at 1-year follow-up.

|  | **Admission** (n=256) | | | | | **Discharge** (n=223) | | | | | **1-year follow-up** (n=313) | | | | |
| --- | --- | --- | --- | --- | --- | --- | --- | --- | --- | --- | --- | --- | --- | --- | --- |
| **HADS item** | **Mis-sing, n** | **Mean** | **SD** | **Floor,**  **%** | **Ceiling,**  **%** | **Mis-sing, n** | **Mean** | **SD** | **Floor,**  **%** | **Ceiling,**  **%** | **Mis-sing, n** | **Mean** | **SD** | **Floor,**  **%** | **Ceiling,**  **%** |
| *Anxiety* |  |  |  |  |  |  |  |  |  |  |  |  |  |  |  |
| 1 | 0 | 0.94 | 0.84 | 33.6 | 4.3 | 0 | 0.86 | 0.80 | 35.9 | 4.0 | 3 | 0.77 | 0.77 | 40.3 | 3.6 |
| 3 | 0 | 0.89 | 0.99 | 45.7 | 9.0 | 0 | 0.70 | 0.86 | 51.6 | 5.4 | 4 | 0.64 | 0.82 | 54.1 | 3.2 |
| 5 | 1 | 1.08 | 0.99 | 34.9 | 10.6 | 0 | 0.83 | 0.89 | 43.1 | 6.3 | 2 | 0.80 | 0.84 | 43.7 | 2.9 |
| 7 | 1 | 0.96 | 0.86 | 33.7 | 5.1 | 0 | 0.85 | 0.81 | 38.6 | 2.7 | 1 | 0.83 | 0.77 | 38.1 | 1.3 |
| 9 | 0 | 0.86 | 0.86 | 38.3 | 6.3 | 1 | 0.65 | 0.73 | 48.2 | 2.3 | 0 | 0.62 | 0.68 | 48.6 | 0.6 |
| 11 | 1 | 1.14 | 0.94 | 29.4 | 8.2 | 0 | 0.95 | 0.90 | 37.7 | 5.8 | 1 | 0.90 | 0.83 | 36.5 | 3.5 |
| 13 | 0 | 0.73 | 0.86 | 48.8 | 5.1 | 0 | 0.60 | 0.82 | 58.3 | 3.6 | 2 | 0.49 | 0.74 | 64.0 | 1.6 |
| *Depression* |  |  |  |  |  |  |  |  |  |  |  |  |  |  |  |
| 2 | 1 | 0.65 | 0.81 | 51.4 | 4.3 | 1 | 0.52 | 0.70 | 58.1 | 1.8 | 2 | 0.69 | 0.77 | 47.3 | 2.3 |
| 4 | 0 | 0.61 | 0.77 | 54.3 | 2.3 | 0 | 0.46 | 0.64 | 61.4 | 0.9 | 3 | 0.57 | 0.71 | 55.5 | 12.6 |
| 6 | 0 | 0.67 | 0.77 | 49.2 | 2.7 | 0 | 0.57 | 0.76 | 55.6 | 3.6 | 2 | 0.61 | 0.76 | 53.7 | 1.9 |
| 8 | 0 | 1.27 | 0.89 | 18.8 | 10.9 | 0 | 1.11 | 0.85 | 23.3 | 8.1 | 0 | 1.22 | 0.85 | 18.5 | 9.0 |
| 10 | 1 | 0.61 | 0.79 | 55.3 | 2.8 | 1 | 0.50 | 0.74 | 62.2 | 2.3 | 2 | 0.50 | 0.71 | 61.4 | 1.3 |
| 12 | 1 | 0.80 | 0.88 | 45.1 | 5.1 | 0 | 0.72 | 0.83 | 48.0 | 4.0 | 2 | 0.77 | 0.84 | 46.3 | 2.9 |
| 14 | 0 | 0.68 | 0.93 | 56.3 | 8.2 | 0 | 0.62 | 0.82 | 55.2 | 4.5 | 2 | 0.54 | 0.80 | 60.5 | 4.5 |

Ceiling, % = proportion of patients scoring at the highest possible item level. Floor, % = proportion of patients scoring at the lowest possible item level. Mean = HADS item values range from 0 to 3 (a higher value indicates more symptoms). Missing, n = number of respondents with missing item value. SD = standard deviation.
