# Supplementary material for: Psychometric characteristics of the Hospital Anxiety and Depression Scale in stroke survivors of working age before and after inpatient rehabilitation
Source: PLoS One. 2024 Aug 26;19(8):e0306754. doi: 10.1371/journal.pone.0306754 (PMC11346913; doi:10.1371/journal.pone.0306754)
Supplement: S3 Table — (DOCX) [file pone.0306754.s005.docx]

**S3 Table.** Multitrait scaling analysis of the Hospital Anxiety and Depression Scale items at admission, discharge, and 1-year follow-up.

|  | **Admission**  (n=256) | | **Discharge**  (n=223) | | **1-year follow-up**  (n=313) | |
| --- | --- | --- | --- | --- | --- | --- |
| *Items* | *Anxiety* | *Depression* | *Anxiety* | *Depression* | *Anxiety* | *Depression* |
| ***Anxiety*** |  |  |  |  |  |  |
| 1 | 0.73 | 0.52 | 0.73 | 0.52 | 0.73 | 0.56 |
| 3 | 0.73 | 0.52 | 0.67 | 0.48 | 0.66 | 0.53 |
| 5 | 0.71 | 0.52 | 0.76 | 0.50 | 0.73 | 0.59 |
| 7 | 0.63 | **0.53** | 0.60 | **0.59** | 0.65 | **0.69** |
| 9 | 0.74 | 0.53 | 0.67 | **0.55** | 0.72 | **0.61** |
| 11 | 0.63 | 0.42 | 0.67 | 0.47 | 0.62 | 0.47 |
| 13 | 0.77 | 0.60 | 0.73 | 0.57 | 0.69 | 0.56 |
| ***Depression*** |  |  |  |  |  |  |
| 2 | 0.34 | 0.54 | 0.37 | 0.66 | 0.55 | 0.70 |
| 4 | **0.50** | 0.62 | 0.52 | 0.69 | 0.54 | 0.73 |
| 6 | **0.61** | 0.63 | **0.55** | 0.66 | 0.59 | 0.71 |
| 8 | **0.50** | 0.49 | **0.57** | 0.45 | **0.54** | 0.53 |
| 10 | 0.40 | 0.54 | **0.43** | 0.51 | **0.54** | 0.63 |
| 12 | 0.44 | 0.67 | 0.46 | 0.75 | 0.63 | 0.78 |
| 14 | **0.45** | 0.53 | **0.50** | 0.61 | **0.52** | 0.60 |

Correlation coefficients between an item and its own subscale are corrected for overlap.

Coefficients ≥0.40 indicate satisfactory item–scale convergent validity. The significance of a difference between two item–scale correlations was determined using the standard error (SE) of the correlation matrix (1/√n). The recommended significance criterion of 2 SE was used: 2 SE at admission = 0.125, at discharge = 0.134, and at 1-year follow-up = 0.113. Coefficients given **in bold** indicate weak item–scale discriminant validity.
