# Supplementary material for: Psychometric characteristics of the Hospital Anxiety and Depression Scale in stroke survivors of working age before and after inpatient rehabilitation
Source: PLoS One. 2024 Aug 26;19(8):e0306754. doi: 10.1371/journal.pone.0306754 (PMC11346913; doi:10.1371/journal.pone.0306754)
Supplement: S4 Table — (DOCX) [file pone.0306754.s006.docx]

**S4 Table.** Confirmatory factor analysis (CFA) loadings of the Hospital Anxiety and Depression Scale items for one- and two-factor models at admission, discharge, and 1-year follow-up.

| **Item** | **Admission** | | | **Discharge** | | | **1-year follow-up** | | |
| --- | --- | --- | --- | --- | --- | --- | --- | --- | --- |
|  | *1 factor* | *2 factors* | | *1 factor* | *2 factors* | | *1 factor* | *2 factors* | |
|  |  | Anx | Dep |  | Anx | Dep |  | Anx | Dep |
| 1 | 0.808 | 0.824 |  | 0.791 | 0.833 |  | 0.776 | 0.855 |  |
| 2 | 0.505 |  | 0.644 | 0.611 |  | 0.791 | 0.759 |  | 0.817 |
| 3 | 0.824 | 0.849 |  | 0.746 | 0.780 |  | 0.733 | 0.779 |  |
| 4 | 0.658 |  | 0.786 | 0.746 |  | 0.859 | 0.797 |  | 0.869 |
| 5 | 0.805 | 0.820 |  | 0.787 | 0.852 |  | 0.790 | 0.846 |  |
| 6 | 0.765 |  | 0.827 | 0.767 |  | 0.806 | 0.806 |  | 0.840 |
| 7 | 0.736 | 0.726 |  | 0.752 | 0.720 |  | 0.815 | 0.769 |  |
| 8 | 0.616 |  | 0.625 | 0.646 |  | 0.550 | 0.662 |  | 0.634 |
| 9 | 0.827 | 0.849 |  | 0.781 | 0.799 |  | 0.814 | 0.857 |  |
| 10 | 0.552 |  | 0.661 | 0.601 |  | 0.640 | 0.745 |  | 0.769 |
| 11 | 0.696 | 0.720 |  | 0.716 | 0.759 |  | 0.646 | 0.708 |  |
| 12 | 0.611 |  | 0.765 | 0.691 |  | 0.857 | 0.845 |  | 0.898 |
| 13 | 0.877 | 0.891 |  | 0.830 | 0.858 |  | 0.768 | 0.819 |  |
| 14 | 0.618 |  | 0.699 | 0.685 |  | 0.743 | 0.727 |  | 0.732 |

Anx = Anxiety. Dep = Depression. All factor loadings are significant (p<0.001).
