# Supplementary material for: Psychometric characteristics of the Hospital Anxiety and Depression Scale in stroke survivors of working age before and after inpatient rehabilitation
Source: PLoS One. 2024 Aug 26;19(8):e0306754. doi: 10.1371/journal.pone.0306754 (PMC11346913; doi:10.1371/journal.pone.0306754)
Supplement: S5 Table — (DOCX) [file pone.0306754.s007.docx]

**S5 Table.** Known-groups validity tests of male–female differences on the Hospital Anxiety and Depression Scale at admission, discharge, and 1-year follow-up.

|  | **Admission**  (n=256) | | **Discharge**  (n=223) | | **1-year follow-up**  (n=313) | |
| --- | --- | --- | --- | --- | --- | --- |
|  | *Anxiety* | *Depression* | *Anxiety* | *Depression* | *Anxiety* | *Depression* |
| *Men*, n (%) | 160 (62.5) | 160 (62.5) | 137 (61.4) | 137 (61.4) | 178 (56.9) | 178 (56.9) |
| Mean | 5.85 | 4.72 | 4.76 | 4.35 | 4.49 | 4.77 |
| SD | 4.56 | 3.88 | 4.28 | 3.82 | 3.95 | 4.18 |
| 95% CI | 5.14–6.56 | 4.11–5.32 | 4.04–5.48 | 3.70–4.99 | 3.90–5.07 | 4.15–5.38 |
| *Women*, n (%) | 96 (37.5) | 96 (37.5) | 86 (38.6) | 86 (38.6) | 135 (43.1) | 135 (43.1) |
| Mean | 7.87 | 6.26 | 6.49 | 4.77 | 5.85 | 5.14 |
| SD | 5.54 | 4.32 | 4.75 | 3.98 | 4.48 | 4.08 |
| 95% CI | 6.75–9.00 | 5.39–7.14 | 5.47–7.51 | 3.91–5.62 | 5.09–6.61 | 4.44–5.83 |
| p-value* | 0.006 | 0.004 | 0.004 | 0.463 | 0.008 | 0.433 |
| Effect size** | 0.40 | 0.38 | 0.38 | 0.11 | 0.32 | 0.09 |

*Men vs. women (Mann–Whitney U-test).

**Effect size (Cohen’s *d*) of the difference between men and women, where 0.00–0.19 = trivial, 0.20–0.49 = small, 0.50–0.79 = medium, and 0.80+ = large.

CI = confidence interval; SD = standard deviation.
