# Supplementary material for: Psychometric characteristics of the Hospital Anxiety and Depression Scale in stroke survivors of working age before and after inpatient rehabilitation
Source: PLoS One. 2024 Aug 26;19(8):e0306754. doi: 10.1371/journal.pone.0306754 (PMC11346913; doi:10.1371/journal.pone.0306754)
Supplement: S6 Table — (DOCX) [file pone.0306754.s008.docx]

**S6 Table.** Response category functioning (Andrich threshold estimates) for Hospital Anxiety and Depression Scale (HADS) anxiety items according to the Rasch partial credit model, at admission, discharge, and 1-year follow-up.

| **HADS**  **anxiety**  **item*** | **Thresholds** between categories** | **Values (logits) at thresholds between categories** | | |
| --- | --- | --- | --- | --- |
|  |  | **Admission**  (n=256) | **Discharge**  (n=223) | **1-yr follow-up**  (n=313) |
| *Item 1* | Category 0–1 | -2.96 | -3.02 | -3.08 |
|  | Category 1–2 | 0.19 | 0.58 | 0.99 |
|  | Category 2–3 | 2.77 | 2.43 | 2.09 |
| Distance 1*** | 0–1/1–2 | 3.15 | 3.60 | 4.07 |
| Distance 2 | 1–2/2–3 | 2.58 | 1.85 | **1.10** |
| *Item 3* | Category 0–1 | -1.77 | -2.07 | -2.22 |
|  | Category 1–2 | 0.12 | 0.51 | 0.20 |
|  | Category 2–3 | 1.65 | 1.56 | 2.02 |
| Distance 1 | 0–1/1–2 | 1.89 | 2.58 | 3.21 |
| Distance 2 | 1–2/2–3 | 1.53 | **1.05** | 1.82 |
| *Item 5* | Category 0–1 | -2.08 | -2.30 | -2.69 |
|  | Category 1–2 | 0.09 | 0.47 | -0.08 |
|  | Category 2–3 | 2.00 | 1.83 | 2.77 |
| Distance 1 | 0–1/1–2 | 2.17 | 2.77 | 2.61 |
| Distance 2 | 1–2/2–3 | 1.91 | **1.36** | 2.85 |
| *Item 7* | Category 0–1 | -2.82 | -2.94 | -3.47 |
|  | Category 1–2 | 0.17 | -0.05 | -0.16 |
|  | Category 2–3 | 2.65 | 2.99 | 3.63 |
| Distance 1 | 0–1/1–2 | 2.99 | 2.89 | 3.31 |
| Distance 2 | 1–2/2–3 | 2.48 | 3.04 | 3.79 |
| *Item 9* | Category 0–1 | -2.67 | -2.89 | -3.58 |
|  | Category 1–2 | 0.85 | 0.67 | 0.15 |
|  | Category 2–3 | 1.83 | 2.22 | 3.44 |
| Distance 1 | 0–1/1–2 | 3.52 | 3.56 | 3.73 |
| Distance 2 | 1–2/2–3 | **0.98** | 1.55 | 3.29 |
| *Item 11* | Category 0–1 | -2.48 | -2.42 | -3.01 |
|  | Category 1–2 | -0.11 | -0.01 | 0.14 |
|  | Category 2–3 | 2.59 | 2.43 | 2.87 |
| Distance 1 | 0–1/1–2 | 2.37 | 2.41 | 3.15 |
| Distance 2 | 1–2/2–3 | 2.70 | 2.44 | 2.73 |
| *Item 13* | Category 0–1 | -2.23 | -1.95 | -2.11 |
|  | Category 1–2 | 0.39 | 0.10 | -0.13 |
|  | Category 2–3 | 1.84 | 1.84 | 2.24 |
| Distance 1 | 0–1/1–2 | 2.62 | 2.05 | 2.24 |
| Distance 2 | 1–2/2–3 | 1.45 | 1.74 | 2.11 |

*Response categories 0, 1, 2, and 3.

**Andrich thresholds between response category 0–1, 1–2, and 2–3.

***The desirable distance between threshold values = 1.4–5.0 logits; distances that do not meet this criterion are given **in bold**.

Fewer than ten observations were noted for category 3 for items 1, 7, 9, 11, and 13 at discharge, and items 5, 7, 9 (both category 2 and category 3), and 13 at follow-up.
