# Supplementary material for: Psychometric characteristics of the Hospital Anxiety and Depression Scale in stroke survivors of working age before and after inpatient rehabilitation
Source: PLoS One. 2024 Aug 26;19(8):e0306754. doi: 10.1371/journal.pone.0306754 (PMC11346913; doi:10.1371/journal.pone.0306754)
Supplement: S7 Table — (DOCX) [file pone.0306754.s009.docx]

**S7 Table.** Response category functioning (Andrich threshold estimates) for Hospital Anxiety and Depression Scale depression items according to the Rasch partial credit model at admission, discharge, and 1-year follow-up.

| **HADS depression item*** | **Thresholds** between categories** | **Values (logits) at thresholds between categories** | | |
| --- | --- | --- | --- | --- |
|  |  | **Admission**  (n=256) | **Discharge**  (n=223) | **1-yr follow-up**  (n=313) |
| *Item 2* | Category 0–1 | -1.66 | -2.16 | -2.70 |
|  | Category 1–2 | 0.87 | 0.49 | 0.41 |
|  | Category 2–3 | 0.79 | 1.67 | 2.29 |
| Distance 1*** | 0–1/1–2 | 2.53 | 2.65 | 3.11 |
| Distance 2 | 1–2/2–3 | **-0.10** | **1.18** | 1.88 |
| *Item 4* | Category 0–1 | -1.72 | -2.44 | -1.21 |
|  | Category 1–2 | 0.30 | 0.38 | 1.21 |
|  | Category 2–3 | 1.42 | 2.06 | NA |
| Distance 1 | 0–1/1–2 | 2.02 | 2.82 | 2.42 |
| Distance 2 | 1–2/2–3 | **1.12** | 1.68 | NA |
| *Item 6* | Category 0–1 | -1.92 | -1.90 | -2.41 |
|  | Category 1–2 | 0.53 | 0.99 | 0.19 |
|  | Category 2–3 | 1.39 | 0.92 | 2.22 |
| Distance 1 | 0–1/1–2 | 2.45 | 2.89 | 2.60 |
| Distance 2 | 1–2/2–3 | **0.86** | **-0.07** | 2.03 |
| *Item* *8* | Category 0–1 | -2.81 | -2.89 | -3.91 |
|  | Category 1–2 | 0.80 | 0.89 | 1.11 |
|  | Category 2–3 | 2.01 | 2.00 | 2.80 |
| Distance 1 | 0–1/1–2 | 3.61 | 3.78 | 5.02 |
| Distance 2 | 1–2/2–3 | **1.21** | **1.11** | 1.69 |
| *Item* *10* | Category 0–1 | -1.58 | -1.78 | -2.26 |
|  | Category 1–2 | 0.24 | 0.25 | 0.10 |
|  | Category 2–3 | 1.33 | 1.52 | 2.16 |
| Distance 1 | 0–1/1–2 | 1.82 | 2.03 | 2.36 |
| Distance 2 | 1–2/2–3 | **1.09** | **1.27** | 2.06 |
| *Item* *12* | Category 0–1 | -1.70 | -2.01 | -2.42 |
|  | Category 1–2 | 0.34 | 0.38 | -0.07 |
|  | Category 2–3 | 1.36 | 1.62 | 2.49 |
| Distance 1 | 0–1/1–2 | 2.04 | 1.63 | 2.35 |
| Distance 2 | 1–2/2–3 | **1.02** | **1.24** | 2.56 |
| *Item* *14* | Category 0–1 | -1.03 | -1.69 | -1.73 |
|  | Category 1–2 | 0.83 | 0.67 | 1.00 |
|  | Category 2–3 | 0.20 | 1.02 | 0.73 |
| Distance 1 | 0–1/1–2 | 1.86 | 2.36 | 2.73 |
| Distance 2 | 1–2/2–3 | **-0.63** | **0.35** | **-0.27** |

*Response categories for HADS items = 0, 1, 2, and 3.

**Andrich thresholds between the response categories 0 to 1, 1 to 2, and 2 to 3.

***The desirable distance between threshold values = 1.4–5.0 logits. Distances that do not meet this criterion are given **in bold**.

Fewer than ten observations were noted for category 3 for items 4, 6, and 10 at admission; for all items at discharge (both category 2 and 3 for item 14); and items 2, 4, 6, 10, and 12 at follow-up.

NA = not applicable; there were no responses in the category.
