# Supplementary material for: Psychometric characteristics of the Hospital Anxiety and Depression Scale in stroke survivors of working age before and after inpatient rehabilitation
Source: PLoS One. 2024 Aug 26;19(8):e0306754. doi: 10.1371/journal.pone.0306754 (PMC11346913; doi:10.1371/journal.pone.0306754)
Supplement: S8 Table — (DOCX) [file pone.0306754.s010.docx]

**S8 Table.** Rasch analysis of differential item functioning (DIF) for Hospital Anxiety and Depression Scale items between the measurement occasions (at admission, discharge, and 1-year follow-up).

|  | **Admission vs.**  **discharge** | | **Admission vs.**  **1-year follow-up** | | **Discharge vs.**  **1-year follow-up** | |
| --- | --- | --- | --- | --- | --- | --- |
|  | DIF contrast | p-value | DIF contrast | p-value | DIF contrast | p-value |
| *Anxiety item* |  |  |  |  |  |  |
| 1 | 0.19 | 0.1987 | -0.07 | 0.6299 | -0.28 | 0.0659 |
| 3 | -0.12 | 0.4049 | -0.28 | 0.0562 | -0.15 | 0.3461 |
| 5 | -0.21 | 0.1488 | -0.31 | 0.0254 | -0.09 | 0.5718 |
| 7 | 0.11 | 0.4632 | 0.05 | 0.7406 | -0.07 | 0.6497 |
| 9 | -0.20 | 0.2014 | -0.31 | 0.0323 | -0.11 | 0.4975 |
| 11 | -0.08 | 0.5756 | -0.21 | 0.1128 | -0.13 | 0.3727 |
| 13 | 0.00 | 1.000 | -0.40 | *0.0087* | -0.40 | *0.0171* |
| *Depression item* |  |  |  |  |  |  |
| 2 | -0.03 | 0.8498 | 0**.50** | *0.0007* | **0.58** | *0.0005* |
| 4 | -0.14 | 0.4211 | 0.26 | 0.0954 | 0.41 | *0.0172* |
| 6 | 0.09 | 0.5640 | 0.22 | 0.1362 | 0.13 | 0.4350 |
| 8 | 0.00 | 1.000 | 0.28 | *0.0294* | 0.27 | 0.0526 |
| 10 | 0.00 | 1.000 | 0.00 | 1.000 | 0.00 | 1.000 |
| 12 | 0.17 | 0.2491 | 0.33 | *0.0200* | 0.16 | 0.3151 |
| 14 | 0.20 | 0.2070 | -0.06 | 0.6810 | -0.29 | 0.0768 |

DIF contrast = <0.5 logits is insignificant, 0.5–1.0 is mild DIF, >1.0 is notable DIF. Values **in bold** show DIF. Significant p-values (<0.05) are given *in italics*.
