# Supplementary material for: Psychometric characteristics of the Hospital Anxiety and Depression Scale in stroke survivors of working age before and after inpatient rehabilitation
Source: PLoS One. 2024 Aug 26;19(8):e0306754. doi: 10.1371/journal.pone.0306754 (PMC11346913; doi:10.1371/journal.pone.0306754)
Supplement: Attachment — Submitted filename: Mustafa Ali_Comments.docx [file pone.0306754.s011.docx]

**Reviewer Name: Mustafa Ali Khalaf**

**Manuscript Title:**

Psychometric characteristics of the Hospital Anxiety and Depression Scale in stroke survivors of working age before and after inpatient rehabilitation.

ID: PONE-D-24-05048

Comments:

1. The introduction is clear, overviewing the main concepts and making the basic case well for the current study.

2. I found the results to be explained clearly and thoroughly. Tables/Figures support the written results.

3. Methods overall are clearly described, including analytic procedures.

4. Author/s should provide the value of MacDonald's Omega as an index of reliability because up-to-date research suggest that Alpha might not be interpreted as an internal consistency coefficient. I would suggest that Cronbach's alpha not be used as a measure of internal consistency. See
Sijtsma, K. (2009). On the use, the misuse, and the very limited usefulness of Cronbach’s alpha. *psychometrika*, *74*, 107-120.‏ <https://doi.org/10.1007/s11336-008-9101-0>

5. For the Item Response Theory (Rasch analysis) and MacDonald's Omega please cite the following article:

Khalaf, M.A., Omara, E.M.N. Rasch analysis and differential item functioning of English language anxiety scale (ELAS) across sex in Egyptian context. *BMC Psychol* **10**, 242 (2022). <https://doi.org/10.1186/s40359-022-00955-w>

6. For anxiety and depression literature, please cite the following article:

Khalaf, M. A., & Al-Said, T. T. (2021). The Egyptian validation study of the resilience scale for adults (RSA) and its utility in predicting depression. *The Open Psychology Journal*, 14, 83-92. https://doi.org/[10.2174/1874350102114010083](http://dx.doi.org/10.2174/1874350102114010083)

7. Do not use abbreviations in the abstract.

8. Author/s wrote: " *frequently used in screening for 60 psychological distress in patients with somatic diseases*". You should provide more than 3 references as long as you said, *"frequently used".*

10. You wrote: " *Patients in the study were registered between 2013 and 2015 at nine rehabilitation centers*". There is a wide gap between the year of data collection and the year of publishing 2024.

11. You should provide the psychometric properties of The HADS that are reported in its original study.

12. For anxiety, please cite the following article:

Khalaf, M.A., Shehata, A. M. (2023). Trust in information sources as a moderator of the impact of COVID-19 anxiety and exposure to information on conspiracy thinking and misinformation beliefs: a multilevel study. *BMC Psychology,* 11, 375. <https://doi.org/10.1186/s40359-023-01425-7>

13. You should provide the differential item functioning (DIF) of the items.

Finally, My decision is: minor correction
