# Supplementary material for: Psychometric characteristics of the Hospital Anxiety and Depression Scale in stroke survivors of working age before and after inpatient rehabilitation
Source: PLoS One. 2024 Aug 26;19(8):e0306754. doi: 10.1371/journal.pone.0306754 (PMC11346913; doi:10.1371/journal.pone.0306754)
Supplement: Attachment — Submitted filename: Response to Reviewers.docx [file pone.0306754.s012.docx]

Dear Academic Editor and Reviewers,

We thank the academic editor and reviewers for thoughtful comments and helpful suggestions for improving the manuscript "Psychometric characteristics of the Hospital Anxiety and Depression Scale in stroke survivors of working age before and after inpatient rehabilitation", ID: PONE-D-24-05048.

Please find below our responses to the comments.

**Journal requirements:**

1. When submitting your revision, we need you to address these additional requirements.

Please ensure that your manuscript meets PLOS ONE's style requirements, including those for file naming. The PLOS ONE style templates can be found at 
<https://journals.plos.org/plosone/s/file?id=wjVg/PLOSOne_formatting_sample_main_body.pdf> and 
<https://journals.plos.org/plosone/s/file?id=ba62/PLOSOne_formatting_sample_title_authors_affiliations.pdf>.

We have reviewed PLOS ONE's style requirements, particularly those for filenames, and believe we have followed the instructions.

2.  note that you have indicated that there are restrictions to data sharing for this study. PLOS only allows data to be available upon request if there are legal or ethical restrictions on sharing data publicly. For more information on unacceptable data access restrictions, please see <http://journals.plos.org/plosone/s/data-availability#loc-unacceptable-data-access-restrictions>.

Before we proceed with your manuscript, please address the following prompts:

a) If there are ethical or legal restrictions on sharing a de-identified data set, please explain them in detail (e.g., data contain potentially identifying or sensitive patient information, data are owned by a third-party organization, etc.) and who has imposed them (e.g., a Research Ethics Committee or Institutional Review Board, etc.). Please also provide contact information for a data access committee, ethics committee, or other institutional body to which data requests may be sent.

b) If there are no restrictions, please upload the minimal anonymized data set necessary to replicate your study findings to a stable, public repository and provide us with the relevant URLs, DOIs, or accession numbers. For a list of recommended repositories, please see
<https://journals.plos.org/plosone/s/recommended-repositories>. You also have the option of uploading the data as Supporting Information files, but we would recommend depositing data directly to a data repository if possible.

We will update your Data Availability statement on your behalf to reflect the information you provide.

We have updated the Data Availability Statement to clarify why there are restrictions on data sharing for this study:

Data from Swedish national quality registers in healthcare cannot be shared publicly as disclosure of data is restricted under Swedish law. Data used in the current study can be requested from the Swedish Register of Rehabilitation Medicine (https://svereh.registercentrum.se) after ethical approval from the Ethical Review Authority (https://etikprovningsmyndigheten.se/en).

3. Please include a copy of Table 6 which you refer to in your text on page 26.

We have included Table 6 in the manuscript (line 359).

**Reviewer #1:**

In this study the authors examined the psychometric properties of the HADS in cohorts of working age stroke survivors. The study has an interesting content. The analyses were done with great care. Nevertheless, I think the manuscript can still be improved by consider the comments raised below:

Major general comment:
* Cisco and colleagues (2012) were able to show in their article, based on a 10-year systematic review, that the HADS has an inability to differentiate between the two constructs anxiety and depression. Coyne and van Sonderen wrote provocatively in 2012 "No further research needed: Abandoning the HADS". On the one hand, I would encourage the authors to justify a little more why they did another analysis (based on this facts/hypotheses). Secondly, I would like to see more reference of their results to other studies/analyses on HADS.

In a comprehensive review of 71 papers, Bjelland et al (2002) concluded that "HADS was found to perform well in assessing symptom severity and incidence of anxiety disorders and depression in both somatic, psychiatric and primary care patients and in the general population." This finding provides support for the HADS as a useful instrument for assessing anxiety and depression symptoms in diverse samples. While we acknowledge opposing opinions, such as those of Coyne and van Sonderen, we believe that there are significantly more studies supporting the validity of the HADS.

Regarding the HADS factor structure, Bjelland et al (2002) concluded that “Most factor analyses demonstrated a two-factor solution in good accordance with the HADS subscales for Anxiety (HADS-A) and Depression (HADS-D), respectively.” However, a more recent review by Cosco et al (2012) found that “Twenty-five of the 50 reviewed studies revealed a two-factor structure, the most commonly found HADS structure. Additionally, five studies revealed unidimensional, 17 studies revealed three-factor, and two studies revealed four-factor structures.” This finding indicates the importance of evaluating the HADS factor structure in different samples to ascertain its appropriateness. This is one reason why we evaluated the factor structure in the current study. However, it should be noted that studies obtaining 3-factor solutions most often present different subfactors for anxiety. These subfactors are highly correlated, and it is questionable whether the division offers any clinical advantages. If the aim is to screen for anxiety symptoms, we believe it is sufficient to measure anxiety as one factor, as confirmed in the current study.

There are several other reasons to continue testing the psychometric properties of the HADS.

Firstly, the methodological techniques for assessing the psychometric properties of self-report questionnaires have been improved over the past decades. Older instruments like the HADS can be tested with new or improved techniques, such as Rasch analysis.

Secondly, developments in quality-of-life research have highlighted the importance of evaluating an instrument's psychometric properties across different disease groups to ensure its appropriateness.

Thirdly, to enhance the measurement of various domains, it is crucial to study individual items to identify areas for improvement within the instrument.

We believe the motives for the current study are adequately described in the Introduction, and no further additions are necessary

Further comments:

Abstract:
The use of subheadings (such as Objects, Methods, Results, ...) could improve readability.
Possibly remove the double mention of the aspects around items 7 and 8 and replace them with another aspect.

Thanks for the suggestion. We have incorporated subheadings into the Abstract and eliminated the duplication of items 7 and 8. In lieu of this, we included results concerning responsiveness in the Abstract (line 17-44). We believe these revisions have enhanced the Abstract.

Introduction:
P. 4: The statement that "the majority of studies..." seems to be correct. I don’t know the whole literature. However, I would expect at least somewhere in the article (here or in the discussion) a reference/comparison to the existing analyses with Rasch (e.g. Tang et al. 2007, or Ayis et al. 2018).

Ayis et al. (2018) is referenced in the discussion with the observation that the study primarily involved elderly stroke patients. Consequently, the findings are not directly comparable to our study, which focuses on stroke patients of working age.

Tang et al. (2007) exclusively examined the HADS depression scale in a small sample (n=100) with a low prevalence of depression disorders (n=17). The vast majority of patients were 65 years or older (n=79). According to Table 2 (Tang et al., 2007), the frequencies for response alternatives 2 and 3 (on the 4-point response scale from 0 to 3) were consistently very low across all 7 depression items, suggesting that the sample size was inadequate and unsuitable for Rasch analysis. Given the small sample and limited variation in response patterns, the reliability of the results is questionable. Consequently, the paper is not cited.

P. 4: Analyses based on both CCT and IRT are performed. A explanation for this is given in the discussion. Possibly paste here.

Thanks for the suggestion. However, we believe this explanation is best suited for the discussion section, as the conclusion was drawn after evaluating the ICC and Rasch results in the current study.

Methods:
P. 5: An imputation of missing values is performed. If I understand the results correctly, then the missing rate was very low. Would it perhaps have been an option to only calculate with already complete data sets? It would be worth considering whether a sentence could be added to the limitations pointing out possible difficulties with imputations.

Thanks for the remark. As described in the Methods section, we utilized the “half-scale” method (and personal mean) to impute missing item values for calculating HADS scale scores. This method is commonly employed for scale scoring and is, for instance, used in the well-known SF-36 Health Survey (Ware et al. 1993). This straightforward imputation approach appears to perform well, particularly when the number of missing items is low (Peyre et al. 2011).

However, for the CCT and Rasch analyses, we utilized observed data without imputation of missing items. Since this was not explicitly stated in the manuscript, we have now included this information in the Methods section (line 113-114).

p. 8: I understand the use of "harder/easier" here and in the rest of the article. The Rasch Model has been used in many analyses in the educational field and there this use of language makes a lot of sense. However, I am not sure if it makes as much sense in the clinical field. A change/adaptation should be considered.

The authors extensively discussed this aspect during the preparing of the manuscript. We recognize the issue of conflicting terminologies regarding describing theta-values for the HADS. Given the psychometric focus of the current study, our approach has been to employ the terms most commonly associated with the Rasch method. We have endeavored to clarify this in the manuscript (lines 171-177): ”a higher difficulty estimate indicates that the item is “harder” to respond to, i.e., more severe anxiety/depression symptoms are required for endorsement, while a lower estimate indicates that the item is “easier,” i.e., less severe anxiety/depression is required for endorsement.” We believe that this explanation is easily comprehensible, enabling readers unfamiliar with Rasch analysis concepts to understand the significance.

p. 8: Only DIF analyses to the measurement times were calculated. It should be considered whether further analyses would be useful, e.g. on gender, age or a clinical parameters.

Thank you for your comment. A key aspect highlighted in our study's findings is the conceptual consistency of HADS over time, i.e., no DIF was observed. This indicates that patients maintain a stable perception of the questions' meaning, unaffected by the rehabilitation efforts. However, we believe that potential DIF concerning factors like gender or age falls beyond the scope of our current investigation, but remains a broader question for the HADS instrument as a whole.

Results:
p. 9: The title includes "survivors of working age". Is there no data on the patients' occupational status?

The aim was to evaluate the psychometric properties of HADS in a sample of stroke survivors of working age; however, we did not intend to investigate occupational status before and after rehabilitation. This is outside the scope of the current study.

p. 10: "Most anxiety symptoms were reported on item 11 ...". I find the wording a bit problematic, especially for item 8. With this statement, we assume that the items measure the underlying construct in a valid way. Your analyses were not able to show this, at least for item

8. I would find it more favorable if, for example, you chose a formulation such as "on item 11 were found the hightest mean values" (just a suggestion).

Thanks for the remark. We agree that this wording is problematic and have changed in accordance with the suggestion (line 224-226).

p. 11: Where do the groupings 8-10 and 11-21 come from? Is this the information from the manual? If so, please cite the source.

Cutoff values for non-cases (score 0-7), doubtful cases (score 8-10), and cases (score 11-21) were presented in the original validation study of the HADS (Zigmond & Snaith, 1983). The same cut-off values are used for both the anxiety and depression scales. We have cited this paper after the first sentence of the paragraph describing HADS in the Methods section (line 105).

p. 12: "possible and probable cases": Reading this gives the impression that a high value indicates a certain disorder. Especially in the area of anxiety, it's not so simple. There is not "one" anxiety disorder. A high score can be an indication of an agoraphopia, a social phobia, a trauma disorder or another disorder.

The HADS was developed to detect states of anxiety and depression among non-psychiatric patients with somatic illnesses. As mentioned in the previous point, Zigmond & Snaith (1983) provided cut-off values that have been widely used in screening for mental disorders in patients with somatic diseases. The concepts of "possible and probable cases" are well-established and regularly employed in the HADS literature. In their comprehensive review, Bjelland et al. (2002) evaluated HADS as a case finder for anxiety disorders and depression. They concluded that “the threshold values identified for optimal balance between sensitivity and specificity showed relatively little variability, and they were very close to 8+, defined as the cut-off for ‘possible cases’ suggested by Zigmond and Snaith in their original paper on HADS.”

The HADS can be used for screening purposes, namely, as a tool to identify individuals with anxiety and depression symptoms who require treatment. For screening purposes, it is not necessary to distinguish between subtypes of anxiety. A specific diagnosis should be established at a personal consultation with a specialist.

Discussion:
p. 20: "measuring mood disorders" - see comment above.

We have modified the sentence and write: “measuring anxiety and depression symptoms” (line 384).

p. 23: "Different analysis techniques.... " Please provide a source for this statement.

We have included a reference in the Discussion section that addresses the combined use of CTT and IRT methods (line 459).

42. Bechger, T. M., Maris, G., Verstralen, H. H., & Béguin, A. A. (2003). Using classical test theory in combination with item response theory. Applied psychological measurement, 27(5), 319-334. https://doi.org/10.1177/0146621603257518 (line 668)

**Reviewer #2:**

Manuscript Title:
Psychometric characteristics of the Hospital Anxiety and Depression Scale in stroke survivors of working age before and after inpatient rehabilitation.
ID: PONE-D-24-05048

Comments:
1. The introduction is clear, overviewing the main concepts and making the basic case well for the current study.

2. I found the results to be explained clearly and thoroughly. Tables/Figures support the written results.

3. Methods overall are clearly described, including analytic procedures.

4. Author/s should provide the value of MacDonald's Omega as an index of reliability because up-to-date research suggest that Alpha might not be interpreted as an internal consistency coefficient. I suggest that Cronbach's alpha should not be used as a measure of internal consistency. See Sijtsma, K. (2009). On the use, the misuse, and the very limited usefulness of Cronbach’s alpha. psychometrika, 74, 107-120.‏ <https://doi.org/10.1007/s11336-008-9101-0>

Thanks for the suggestion. We have incorporated MacDonald's omega coefficients in Table 6, which displays measures of reliability at the three measurement occasions. Unfortunately, Table 6 was missing from the submitted manuscript, but it is now included (line 360). We have also updated the Abstract (line 32), Methods (line 117), Results (line 353) and Discussion (line 438, 445) sections accordingly.

We have added a reference to omega in the Methods section (line 118):

22. Hayes, A. F., & Coutts, J. J. (2020). Use omega rather than Cronbach’s alpha for estimating reliability. But…. Communication Methods and Measures, 14(1), 1-24. https://doi.org/10.1080/19312458.2020.1718629 (line 606)

Since most HADS validation papers present alpha, we prefer to retain alpha to facilitate comparisons with other studies. Additionally, comparing alpha and omega estimates is valuable. In the current study, alpha and omega coefficients were identical for the anxiety and depression scales at all three measurement occasions, maintaining consistency in the interpretation of internal consistency reliability.

5. For the Item Response Theory (Rasch analysis) and MacDonald's Omega please cite the following article:
Khalaf, M.A., Omara, E.M.N. Rasch analysis and differential item functioning of English language anxiety scale (ELAS) across sex in Egyptian context. BMC Psychol 10, 242 (2022). <https://doi.org/10.1186/s40359-022-00955-w>

Thank you for this suggestion. Although we agree that adding MacDonald's Omega is relevant for the manuscript, we could not find any information in the suggested article regarding MacDonald's Omega when applying Item Response Theory (Rasch analysis). Thus, we decided not to cite the suggested article.

6. For anxiety and depression literature, please cite the following article:
Khalaf, M. A., & Al-Said, T. T. (2021). The Egyptian validation study of the resilience scale for adults (RSA) and its utility in predicting depression. The Open Psychology Journal, 14, 83-92. <https://doi.org/10.2174/1874350102114010083>

The suggested article is a validation study of a translated version of a scale measuring resilience, in a population of university students. Since the reference does not concern HADS, nor patients with stroke, or any other information on anxiety and depression of relevance to our manuscript, we found no justification in citing the suggested article.

7. Do not use abbreviations in the abstract.

We have used the abbreviation HADS five times in the abstract. HADS is an accepted abbreviation that the reader is likely to be familiar with. We think it is justified to use this abbreviation because it facilitates readability. By using the abbreviation, the information in the abstract, which is limited to 250 words, can be used in a more efficient way.

8. Author/s wrote: " frequently used in screening for 60 psychological distress in patients with somatic diseases". You should provide more than 3 references as long as you said, "frequently used".

We have cited review papers that encompassed a large number of studies. Bjelland et al (2002) reviewed 71 papers, and in the first sentence of the Results section, they conclude: “Most studies using HADS have been done on selected samples of patients with cancer or other somatic illnesses.” We believe this conclusion provides sufficient support that HADS is frequently used for assessing psychological distress in patients with somatic diseases, and no additional references are needed to support this point.

10. You wrote: " Patients in the study were registered between 2013 and 2015 at nine rehabilitation centers". There is a wide gap between the year of data collection and the year of publishing 2024.

We have used available HADS data from a Swedish national quality register. We do not consider the time of data collection to affect the psychometric results.

11. You should provide the psychometric properties of The HADS that are reported in its original study.

HADS was presented by Zigmond & Snaith in 1983, but the original study contains limited psychometric data. Numerous subsequent studies have examined the psychometric properties of the HADS. We have cited Zigmond & Snaith (1983) in the description of the HADS instrument in the Method section. However, there are no other data in the original paper that directly correspond to results in the current study.

12. For anxiety, please cite the following article:
Khalaf, M.A., Shehata, A. M. (2023). Trust in information sources as a moderator of the impact of COVID-19 anxiety and exposure to information on conspiracy thinking and misinformation beliefs: a multilevel study. BMC Psychology, 11, 375. <https://doi.org/10.1186/s40359-023-01425-7>

Thank you for the suggested reference. However, the article examines the relationship between exposure to information sources, trust, conspiracy beliefs, misinformation beliefs, and COVID-19 anxiety among healthy adults. This subject matter seems largely disconnected from the anxiety our stroke patients may experience, especially considering that our data was collected before the COVID-19 pandemic.

13. You should provide the differential item functioning (DIF) of the items.

DIF for items are presented in the Supporting information document: S8 Table.docx “Rasch analysis of differential item functioning (DIF) for Hospital Anxiety and Depression Scale items between the measurement occasions (at admission, discharge, and 1-year follow-up).”

Finally, My decision is: minor correction
