# Supplementary material for: Psychometric characteristics of the Hospital Anxiety and Depression Scale in stroke survivors of working age before and after inpatient rehabilitation
Source: PLoS One. 2024 Aug 26;19(8):e0306754. doi: 10.1371/journal.pone.0306754 (PMC11346913; doi:10.1371/journal.pone.0306754)
Supplement: Attachment — Submitted filename: Response to Reviewers.docx [file pone.0306754.s013.docx]

PONE-D-24-05048R1

Psychometric characteristics of the Hospital Anxiety and Depression Scale in stroke survivors of working age before and after inpatient rehabilitation

Dear Academic Editor and Reviewers,

We thank the academic editor and reviewers for helpful suggestions for improving our manuscript "Psychometric characteristics of the Hospital Anxiety and Depression Scale in stroke survivors of working age before and after inpatient rehabilitation".

Please find below our responses.

Journal Requirements:

Please review your reference list to ensure that it is complete and correct. If you have cited papers that have been retracted, please include the rationale for doing so in the manuscript text, or remove these references and replace them with relevant current references. Any changes to the reference list should be mentioned in the rebuttal letter that accompanies your revised manuscript. If you need to cite a retracted article, indicate the article’s retracted status in the References list and also include a citation and full reference for the retraction notice.

**Authors' response:**

We have gone through the reference list and checked all references. We have supplemented some references with DIO information and added PMID information where available. We have not used references that have been retracted.

[Note: HTML markup is below. Please do not edit.]

Reviewers' comments:

Reviewer's Responses to Questions

**Comments to the Author**

1. If the authors have adequately addressed your comments raised in a previous round of review and you feel that this manuscript is now acceptable for publication, you may indicate that here to bypass the “Comments to the Author” section, enter your conflict of interest statement in the “Confidential to Editor” section, and submit your "Accept" recommendation.

Reviewer #1: (No Response)

Reviewer #2: (No Response)

2. Is the manuscript technically sound, and do the data support the conclusions?

The manuscript must describe a technically sound piece of scientific research with data that supports the conclusions. Experiments must have been conducted rigorously, with appropriate controls, replication, and sample sizes. The conclusions must be drawn appropriately based on the data presented.

Reviewer #1: Yes

Reviewer #2: Partly

3. Has the statistical analysis been performed appropriately and rigorously?

Reviewer #1: Yes

Reviewer #2: Yes

4. Have the authors made all data underlying the findings in their manuscript fully available?

The [PLOS Data policy](https://eur02.safelinks.protection.outlook.com/?url=http%3A%2F%2Fwww.plosone.org%2Fstatic%2Fpolicies.action%23sharing&data=05%7C02%7Cjan.karlsson2%40regionorebrolan.se%7Cce4df3055bf84b9066bc08dc89236745%7Caece5b1982274c2782181aea120ec062%7C0%7C0%7C638536032366653837%7CUnknown%7CTWFpbGZsb3d8eyJWIjoiMC4wLjAwMDAiLCJQIjoiV2luMzIiLCJBTiI6Ik1haWwiLCJXVCI6Mn0%3D%7C0%7C%7C%7C&sdata=GJynLJrPRFDhU3e2C%2Fek%2FVGF%2FyEnWyWpX6ihhI8rpaA%3D&reserved=0) requires authors to make all data underlying the findings described in their manuscript fully available without restriction, with rare exception (please refer to the Data Availability Statement in the manuscript PDF file). The data should be provided as part of the manuscript or its supporting information, or deposited to a public repository. For example, in addition to summary statistics, the data points behind means, medians and variance measures should be available. If there are restrictions on publicly sharing data—e.g. participant privacy or use of data from a third party—those must be specified.

Reviewer #1: Yes

Reviewer #2: Yes

5. Is the manuscript presented in an intelligible fashion and written in standard English?

PLOS ONE does not copyedit accepted manuscripts, so the language in submitted articles must be clear, correct, and unambiguous. Any typographical or grammatical errors should be corrected at revision, so please note any specific errors here.

Reviewer #1: Yes

Reviewer #2: No

6. Review Comments to the Author

Please use the space provided to explain your answers to the questions above. You may also include additional comments for the author, including concerns about dual publication, research ethics, or publication ethics. (Please upload your review as an attachment if it exceeds 20,000 characters)

Reviewer #1: Reviewer #1 _ Revision 2.0
Dear author, thank you very much for the comprehensive answers and the changes that have already been implemented. There are currently two areas that remain open for me, for which I hope a simple solution/compromise can be found.

1.) For the most part, I can support your comments. And I am a great advocate of further psychometric studies. I have read and commented on your manuscript and remain convinced that the introduction is a little short on existing analyses of the HADS and the need for this study. You described this very clearly in our brief exchange - but I feel this is somewhat lacking in the manuscript. My suggestion would therefore be that you add 4-5 sentences in the introduction. I think that there are readers (like me) who would like to have a little more information about the state of research and your reasons for the study.

Comment 1.0: “Cisco and colleagues (2012) were able to show in their article, based on a 10-year systematic review, that the HADS has an inability to differentiate between the two constructs anxiety and depression. Coyne and van Sonderen wrote provocatively in 2012 "No further research needed: Abandoning the HADS". On the one hand, I would encourage the authors to justify a little more why they did another analysis (based on this facts/hypotheses). Secondly, I would like to see more reference of their results to other studies/analyses on HADS.”
Respond: “In a comprehensive review of 71 papers, Bjelland et al (2002) concluded that "HADS was found to perform well in assessing symptom severity and incidence of anxiety disorders and depression in both somatic, psychiatric and primary care patients and in the general population." This finding provides support for the HADS as a useful instrument for assessing anxiety and depression symptoms in diverse samples. While we acknowledge opposing opinions, such as those of Coyne and van Sonderen, we believe that there are significantly more studies supporting the validity of the HADS. Regarding the HADS factor structure, Bjelland et al (2002) concluded that “Most factor analyses demonstrated a two-factor solution in good accordance with the HADS subscales forAnxiety (HADS-A) and Depression (HADS-D), respectively.” However, a more recent review by Cosco et al (2012) found that “Twenty-five of the 50 reviewed studies revealed a two-factor structure, the most commonly found HADS structure. Additionally, five studies revealed unidimensional, 17 studies revealed three-factor, and two studies revealed four factor structures.” This finding indicates the importance of evaluating the HADS factor structure in different samples to ascertain its appropriateness. This is one reason why we evaluated the factor structure in the current study. However, it should be noted that studies obtaining 3-factor solutions most often present different subfactors for anxiety. These subfactors are highly correlated, and it is questionable whether the division offers any clinical advantages. If the aim is to screen for anxiety symptoms, we believe it is sufficient to measure anxiety as one factor, as confirmed in the current study. There are several other reasons to continue testing the psychometric properties of the HADS. Firstly, the methodological techniques for assessing the psychometric properties of self-report questionnaires have been improved over the past decades. Older instruments like the HADS can be tested with new or improved techniques, such as Rasch analysis. Secondly, developments in quality-of-life research have highlighted the importance of evaluating an instrument's psychometric properties across different disease groups to ensure its appropriateness. Thirdly, to enhance the measurement of various domains, it is crucial to study individual items to identify areas for improvement within the instrument. We believe the motives for the current study are adequately described in the Introduction, and no further additions are necessary.”

**Authors' response:**

Thanks for this helpful suggestion. We have added two paragraphs to the Introduction that briefly describe the background and motivations for the current psychometric study of the HADS (lines 74-89).

2.) The second area concerns the DIF analyses. It is perfectly understandable to focus and I am not asking for additional analyses in this manuscript. However, I would like you to add a sentence that further analyses on DIF (test fairness) are possible and can provide further insights. The sentence can simply be added somewhere in the discussion. There are some studies (not on stroke patients, I assume), that may offer certain hypotheses for possible DIF problems (gender, age, etc.) (e.g. Cameron et al. 2013).

Comment 1.0: “Only DIF analyses to the measurement times were calculated. It should be considered whether further analyses would be useful, e.g. on gender, age or a clinical parameters.”
Respond: “Thank you for your comment. A key aspect highlighted in our study's findings is the conceptual consistency of HADS over time, i.e., no DIF was observed. This indicates that patients maintain a stable perception of the questions' meaning, unaffected by the rehabilitation efforts. However, we believe that potential DIF concerning factors like gender or age falls beyond the scope of our current investigation, but remains a broader question for the HADS instrument as a whole.”

**Authors' response:**

Thanks for the suggestion. We have added a sentence to the discussion of DIF (lines 448-450). We have also included the suggested reference (Cameron, 2013).

Reviewer #2: Some of my comments were not Done at all. I advise authors to do them. I think they will increase the quality of the manuscript.

**Authors' response:**

Dear Reviewer #2, we sincerely appreciate your valuable comments and suggestions to improve our manuscript. In our previous response, we have thoroughly addressed all your feedback, making changes where appropriate. In cases where we did not accommodate proposed changes, we have provided clear justifications. For instance, some article suggestions were not included as they pertain to topics outside the scope of our study.

7. PLOS authors have the option to publish the peer review history of their article ([what does this mean?](https://eur02.safelinks.protection.outlook.com/?url=https%3A%2F%2Fjournals.plos.org%2Fplosone%2Fs%2Feditorial-and-peer-review-process%23loc-peer-review-history&data=05%7C02%7Cjan.karlsson2%40regionorebrolan.se%7Cce4df3055bf84b9066bc08dc89236745%7Caece5b1982274c2782181aea120ec062%7C0%7C0%7C638536032366653837%7CUnknown%7CTWFpbGZsb3d8eyJWIjoiMC4wLjAwMDAiLCJQIjoiV2luMzIiLCJBTiI6Ik1haWwiLCJXVCI6Mn0%3D%7C0%7C%7C%7C&sdata=ogmYxyWLAxSy5VI7p3smVzWeb5xpcxensNjRDSeepn0%3D&reserved=0)). If published, this will include your full peer review and any attached files.


If you choose “no”, your identity will remain anonymous but your review may still be made public.


**Do you want your identity to be public for this peer review?** For information about this choice, including consent withdrawal, please see our [Privacy Policy](https://eur02.safelinks.protection.outlook.com/?url=https%3A%2F%2Fwww.plos.org%2Fprivacy-policy&data=05%7C02%7Cjan.karlsson2%40regionorebrolan.se%7Cce4df3055bf84b9066bc08dc89236745%7Caece5b1982274c2782181aea120ec062%7C0%7C0%7C638536032366653837%7CUnknown%7CTWFpbGZsb3d8eyJWIjoiMC4wLjAwMDAiLCJQIjoiV2luMzIiLCJBTiI6Ik1haWwiLCJXVCI6Mn0%3D%7C0%7C%7C%7C&sdata=bgsJlOIDE7X8sChFwnq7f9GLTeT0NX8ZdEO5fJ71adw%3D&reserved=0).

Reviewer #1: No

Reviewer #2: No
